# Role of neutrophil to lymphocyte and monocyte to lymphocyte ratios in the diagnosis of bacterial infection in patients with fever

**DOI:** 10.1007/s15010-016-0972-1

**Published:** 2016-12-19

**Authors:** Are Naess, Siri Saervold Nilssen, Reidun Mo, Geir Egil Eide, Haakon Sjursen

**Affiliations:** 1 Department of Clinical Science, Haukeland University Hospital, University of Bergen, N-5021, Bergen, Norway; 2 Department of Medicine, Haukeland University Hospital, Bergen, Norway; 3 Centre for Clinical Research, Haukeland University Hospital, Bergen, Norway; 4 Lifestyle Epidemiology Research Group, Department of Global Public Health and Primary Care, University of Bergen, Bergen, Norway

**Keywords:** Fever, Infections, Neutrophil:lymphocyte ratio (NLR), Monocyte:lymphocyte ratio (MLR)

## Abstract

**Purpose:**

To study the role of the neutrophil:lymphocyte ratio (NLR) and monocyte:lymphocyte ratio (MLR) in discriminating between different patient groups hospitalized for fever due to infection and those without infection.

**Methods:**

For 299 patients admitted to hospital for fever with unknown cause, a number of characteristics including NLR and MLR were recorded. These characteristics were used in a multiple multinomial regression analysis to estimate the probability of a final diagnostic group of bacterial, viral, clinically confirmed, or no infection.

**Results:**

Both NLR and MLR significantly predicted final diagnostic group. Being highly correlated, however, both variables could not be retained in the same model. Both variables also interacted significantly with duration of fever. Generally, higher values of NLR and MLR indicated larger probabilities for bacterial infection and low probabilities for viral infection. Patients with septicemia had significantly higher NLR compared to patients with other bacterial infections with fever for less than one week. White blood cell counts, neutrophil counts, and C-reactive proteins did not differ significantly between septicemia and the other bacterial infection groups.

**Conclusions:**

NLR is a more useful diagnostic tool to identify patients with septicemia than other more commonly used diagnostic blood tests. NLR and MLR may be useful in the diagnosis of bacterial infection among patients hospitalized for fever.

## Introduction

The commonly observed, but not absolute, association between bacterial infection and neutrophil leukocytosis, and between viral infection and lymphocytosis, has long been established.

The neutrophil:lymphocyte ratio (NLR) in peripheral blood, accordingly, has been suggested to be useful for the discrimination between these types of infection [1–3], and also to predict the outcome of infection [4–7].

However, studies have also shown changes in the NLR in a plenitude of non-infectious conditions, including cardiovascular [8–10] and malignant [11] disease, and related to mortality in patients with sepsis [12] and chronic obstructive pulmonary disease (COPD) [13] as well as in critical [14] and malignant [15, 16] illness.

The monocyte:lymphocyte ratio (MLR) has been used in some studies to identify patients at risk for influenza, malaria and tuberculosis [17–21]. Interestingly, in a study of influenza-like illness, Cunha et al. [22] found influenza A and human parainfluenza virus type 3 infection to be associated with MLR > 2, as opposed to infections with human metapneumovirus, rhinovirus/enterovirus, and respiratory syncytial virus, all with MLR < 2.

In a retrospective study of patients hospitalized for fever without a known origin [23], we found the NLR to be higher in patients with fever due to bacterial infections than in those with viral infection.

We now extend the analyses to investigate whether the NLR or the MLR could be more useful to differentiate between patients hospitalized with fever due to infection (bacterial and viral) and those with fever due to non-infectious causes, and if the duration of pre-hospital fever made any difference.

Patients with fever represent a diagnostic challenge to the clinician, and these ratios, easily derived from commonly performed peripheral blood differential counts, could, conceivably, be useful for discriminating between the different causes of fever and between different causes of infections.

## Patients and measurements

The patient groups have been described in a preceding paper [23]. Briefly, 299 patients hospitalized at Haukeland University Hospital, Bergen, Norway for fever without any causal diagnosis were classified according to the duration of pre-hospital fever and their final diagnosis:

*Bacterial infection* One hundred and fifty patients with a diagnosis of bacterial infection supported by microbiology, serology, or radiology of which 69 had pneumonia, 30 urinary tract infection, and 27 had septicemia.

*Viral infection* Fourteen patients with a diagnosis of viral infection supported by microbiology, serology or radiology. Of these, nine suffered from infectious mononucleosis.

*Clinically diagnosed infection* Sixty-six patients with a typical clinical picture of infection, but not supported by microbiology, serology, or radiology.

*No infection* Twenty-nine patients whose fever was found to be caused by non-infectious conditions; eight with immunological and five with malignant disease.

*No diagnosis* Twelve patients without any diagnosis explaining their fever.

Twenty-six immunocompromised or immunosuppressed patients (24 with solid organ or bone marrow transplantation and two with HIV infection) have been included. Patients with leukemia were excluded because of abnormal test results connected with their underlying disease (abnormal white blood cell counts (WBC)).

The following characteristics were registered at admission: age, gender, temperature, and C-reactive protein (CRP). WBC and differential cell counts were obtained by Cell-Dyn 4000 (Abbott Laboratories, North Chicago, IL, USA) and Advia 120 (Siemens, Erlangen, Germany) hematology systems.

## Statistics

For descriptive statistics we use the mean, median, interquartile range (IQR), count, and percentage. For estimating correlation we used both Pearson’s R and Spearman’s rho.

Comparison between independent groups was done with the Wilcoxon-Mann–Whitney test as the variables had highly right-skewed distributions.

A multiple multinomial logistic regression analysis [24] was performed to model the probability of getting a diagnosis in each of four diagnostic groups (bacterial infection, viral infection, clinically diagnosed infection, and no infection), dependent on NLR and MLR and adjusted for the potential predictors age, gender, duration of fever before admission, temperature at admission, WBC count, NLR and MLR. The impact of the various predictors was tested by the likelihood ratio (LR) test, and the results are given by adjusted odds ratios (OR) with 95% confidence interval (CI). Finally, interactions between NLR and fever group and between MLR and fever group were tested. Probabilities for getting a diagnosis in each of the four diagnostic groups were estimated from the model. ROC curves were constructed to show sensitivity and specificity of NLR and MLR with respect to bacterial infection. A significance level of 0.05 was used for all statistical tests. All statistical analyses were done using SPSS 22.

## Results

In patients hospitalized for fever, we found NLR and MLR to be significantly higher in those with bacterial infection than in patients without infection and lower in those with viral infection (Table 1).

**Table 1:** Neutrophil:lymphocyte ratio and monocyte:lymphocyte ratio of patients with bacterial, viral, or clinically diagnosed infections as compared with patients with fever due to non-infectious conditions for 266 patients

Ratio infection group	*n*	Mean	SE	Median	Q_1_	Q_3_	*p* value^a^
NLR							
Bacterial	150	12.23	0.98	7.94	4.47	15.02	<0.001
Viral	14	2.41	0.75	0.63	0.31	3.98	0.010
Clinically diagnosed	66	7.87	1.33	4.27	2.45	8.60	0.313
No infection	36	5.02	0.67	3.78	2.00	7.07	Reference
MLR							
Bacterial^b^	149	0.89	0.06	0.70	0.43	1.03	<0.001
Viral	14	0.25	0.09	0.14	0.05	0.30	0.005
Clinically diagnosed	66	0.71	0.08	0.52	0.31	0.90	0.017
No infection	36	0.46	0.06	0.35	0.20	0.60	Reference

*SE* standard error of the mean, *Q*_*1*_ 1st quartile, *Q*_*3*_ 3rd quartile, *NLR* neutrophil:lymphocyte ratio, *MLR* monocyte:lymphocyte ratio

^a^ *p* values from Wilcoxon–Mann–Whitney test for comparison with the no infection group

^b^One patient had missing MLR

This was more pronounced in patients with fever of less than one week’s duration. Patients with bacterial infection and fever for less than one week had, indeed, significantly higher NLR and MLR than patients with bacterial infection and fever lasting for 1–3 weeks before hospitalization (Table 2).

**Table 2:** Neutrophil:lymphocyte ratio and monocyte:lymphocyte ratio of patients with fever due to bacterial infection for less than 7 days or between 7 and 21 days before hospitalization for 131 patients with fever

Ratio fever group	*n*	Mean	SE	Median	*p* value^a^
NLR					0.005
Fever <7 days	110	13.29	1.23	8.43	
Fever 7–21 days	21	7.21	1.63	4.33	
MLR					0.001
Fever <7 days^b^	109	0.97	0.07	0.71	
Fever 7–21 days	21	0.51	0.08	0.41	

*SE* standard error of the mean, *NLR* neutrophil:lymphocyte ratio, *MLR* monocyte:lymphocyte ratio

^a^Comparing the two fever groups by exact Wilcoxon–Mann–Whitney test

^b^One patient had missing MLR

Among patients with fever of less than one week’s duration, patients with septicemia had significantly higher NLR compared to patients with other bacterial infections (Table 3).

**Table 3:** Comparison of neutrophil:lymphocyte ratio, monocyte:lymphocyte ratio and other variables between septicaemia and other bacterial infections^a^ for 121 patients with pre-hospital fever for less than 7 days

Variable	Statistic	Diagnosis					Total	MWM test^a^ *p* value
		Septicemia	Pneumonia	Pyelo nephritis	Lower UTI	Other infection		
NLR	*n*	18	50	7	15	19	109	
	Mean	23.17	11.01	8.73	13.97	10.79	13.24	
	SE	4.40	1.38	1.36	3.82	2.42	1.23	
	Median	15.69	7.91	8.18	7.88	8.00	8.42	0.006
MLR	*n*	18	50	7	15	18	108	
	Mean	1.38	0.89	1.17	0.99	0.65	0.96	
	SE	0.22	0.11	0.24	0.19	0.06	0.07	
	Median	1.21	0.70	0.90	0.59	0.73	0.71	0.073
WBC	*n*	18	50	7	15	30	120	
	Mean	13.4	14.6	15.0	15.1	13.4	14.2	
	SE	1.5	1.0	2.1	1.5	1.2	0.6	
	Median	11.4	13.4	13.5	12.9	13.5	13.1	0.559
Neutrophils	*n*	18	50	7	15	20	110	
	Mean	12.5	11.8	12.4	11.7	11.6	11.9	
	SE	2.0	0.9	2.0	1.4	1.7	0.6	
	Median	10.3	10.8	10.3	10.8	10.3	10.5	0.677
Lymphocytes	*n*	18	50	7	15	19	109	
	Mean	0.8	1.5	1.5	1.4	1.4	1.3	
	SE	0.1	0.1	0.1	0.2	0.2	0.1	
	Median	0.7	1.3	1.7	1.5	1.3	1.3	<0.001
Monocytes	*n*	18	50	7	15	18	108	
	Mean	0.8	1.0	1.5	1.0	0.8	0.1	
	SE	0.1	0.1	0.2	0.1	0.1	0.0	
	Median	0.7	1.0	1.4	1.0	0.7	1.0	0.078
CRP	*n*	18	50	7	15	31	121	
	Mean	134.9	168.7	261.4	129.3	108.3	148.7	
	SE	25.4	19.6	29.7	23.1	16.0	10.8	
	Median	125.5	137.0	249.0	142.0	77.0	135.0	0.615

*NLR* neutrophil:lymphocyte ratio, *MLR* monocyte:lymphocyte ratio, *WBC* white blood cell count, *CRP* C-reactive protein, *SE* standard error, *UTI* urinary tract infection

^a^Wilcoxon–Mann–Whitney test for septicaemia versus the other bacterial infections combined

**Fig. 1:**
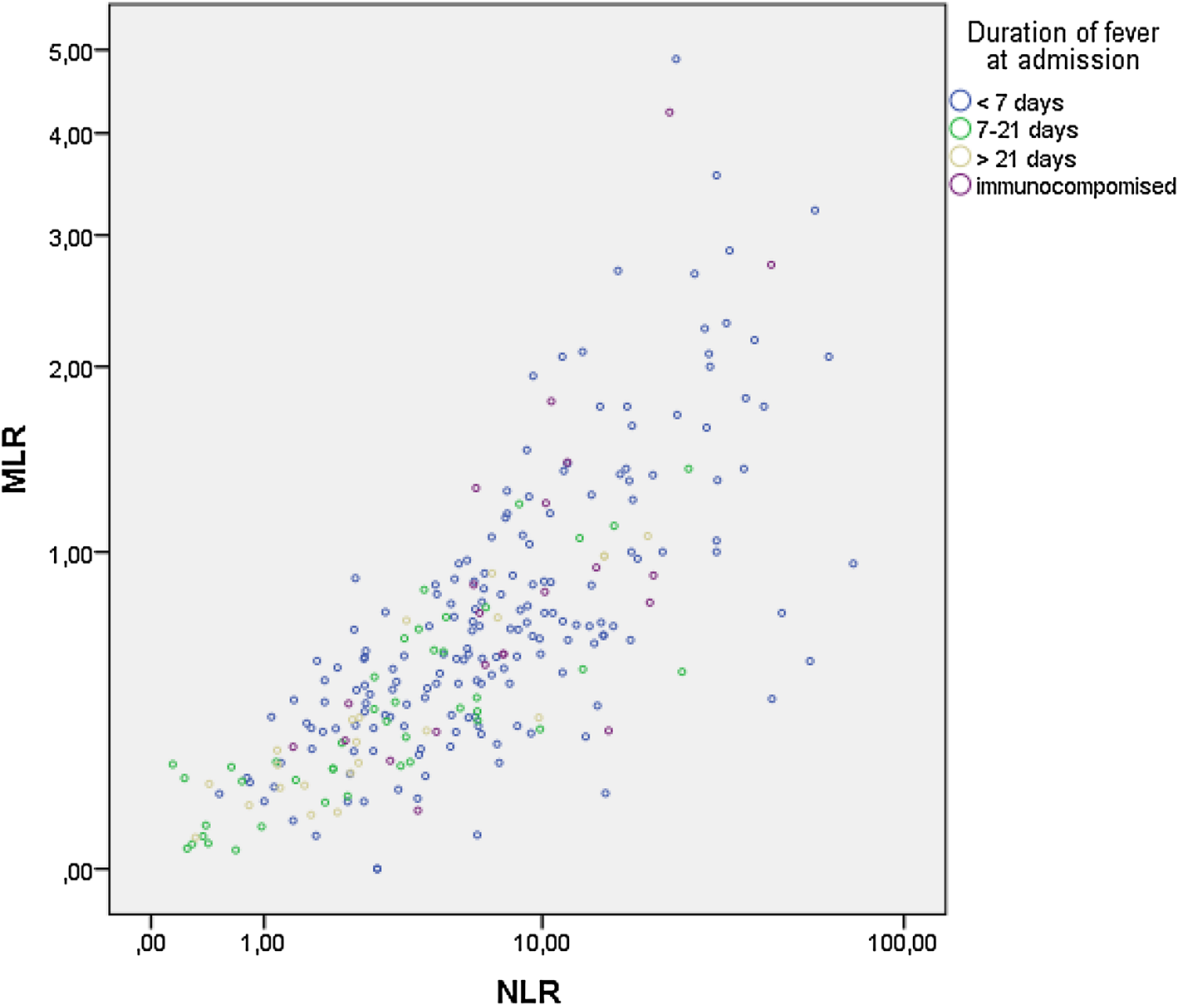
Scattergram on log_10_ *scales* of monocyte:lymphocyte ratio (MLR) versus neutrophil:lymphocyte ratio (NLR) (Pearson’s R: 0.63, Spearman’s rho = 0.78) for 265 patients hospitalized for fever with unknown diagnosis

In multinomial regression unadjusted and adjusting only for age and gender, both NLR and MLR were significant predictors of the infection group (*p* < 0.001 for both). However, adjusting the effects of NLR and MLR for each other gives only borderline significant effects (*p* = 0.095 and 0.055, respectively, adjusted for age and gender; *p* = 0.040 and 0.054 unadjusted for age and sex) as they are highly correlated (Spearman’s rho = 0.78, *p* < 0.001). In Fig. 1, the relationship is shown on a log_10_ scale. For this reason, in further analyses, it was decided not to include NLR and MLR simultaneously in the same model.

Then, in a multiple multinomial regression of NLR and MLR, respectively, adjusting for age, gender, duration of fever, temperature, WBC count, and CRP group, both were found to be statistically significant (*p* = 0.003 and *p* = 0.001). Finally, testing interaction between NLR and fever duration group gave *p* = 0.005 and between MLR and fever duration group gave *p* = 0.001. Table 4 gives the final results with effects of NLR within each fever duration group, and Table 5 likewise for MLR.

**Table 4:** Multiple multinomial logistic regression analysis of final infection group on neutrophil:lymphocyte ratio (NLR) in peripheral blood adjusting for age, temperature and laboratory values for 265^a^ patients with fever without any diagnosis suggesting the cause of the fever admitted to the Medical Department, Haukeland University Hospital, Bergen, Norway from July 1st, 2001 until June 30th, 2004. Data from patients with bacterial, viral, or clinically diagnosed infection have been compared with those from patients without infection (*n* = 36)

Infection group:	Bacterial (*n* = 150)		Viral (*n* = 14)		Clinically diagnosed (*n* = 65)		LR test
Predictors at admittance	OR	95% CI	OR	95% CI	OR	95% CI	*p* value
Age per 10 years	1.15	(0.94, 1.41)	0.92	(0.60, 1.41)	1.00	(0.80, 1.23)	0.194
Female	1.74	(0.69, 4.35)	1.24	(0.22, 6.86)	1.06	(0.41, 2.74)	0.436
Duration of fever							0.020
I (<7 days)	1.00	Reference	1.00	Reference	1.00	Reference	
II (7–21 days)	0.79	(0.17, 3.67)	146.95	(2.06, 10486.46)	0.82	(0.15, 4.41)	
III (>21 days)	0.15	(0.02, 1.06)	n.c.	n.c.	0.21	(0.01, 3.29)	
IV (i.c.)	1.08	(0.04, 13.98)	2.41	(0.03, 202.64)	1.12	(0.06, 21.36)	
Temperature (°C)	1.99	(1.27, 3.10)	2.35	(0.98, 5.63)	1.69	(1.06, 2.69)	0.001
WBC count (×10^9^/L)	1.08	(0.98, 1.20)	1.03	(0.86, 1.23)	0.98	(0.88, 1.09)	0.015
CRP group							0.001
>100 mg/L	4.92	(1.09, 22.24)	0.36	(0.02, 5.16)	2.25	(0.44, 11.46)	
11–100 mg/L	1.52	(0.38, 5.97)	0.31	(0.04, 2.48)	2.69	(0.65, 11.14)	
0–10 mg/L	1.00	Reference	1.00	Reference	1.00	Reference	
NLR × Duration of fever^b^							<0.001
I (<7 days)	1.02	(0.93,1.11)	0.81	(0.56, 1.17)	1.02	(0.94, 1.12)	
II (7–21 days)	0.98	(0.79,1.21)	0.00	(0.00, 3.05)	0.91	(0.67, 1.25)	
III (>21 days)	1.12	(0.84,1.50)	0.00	(0.00, 0.00)	0.82	(0.35, 1.93)	
IV (i.c.)	1.07	(0.76, 1.51)	0.93	(0.55, 1.54)	0.94	(0.65, 1.37)	

*LR* likelihood ratio, *OR* odds ratio, *CI* confidence interval, *WBC* white blood cell, *CRP* C-reactive protein, *i.c.* immunocompromised patients, *n.c.* not computable

^a^Altogether 299 patients were available for analysis, but 34 had missing data on one or more variables, leaving 265 patients for the multiple regression analysis

^b^Test of interaction: *p* = 0.005

**Table 5:** Multiple multinomial logistic regression analysis of final infection group on monocyte:lymphocyte ratio (MLR) in peripheral blood adjusting for age, temperature and laboratory values for 264^a^ patients with fever without any diagnosis suggesting the cause of the fever admitted to the Medical Department, Haukeland University Hospital, Bergen, Norway from July 1st, 2001 until June 30th, 2004. Data from patients with bacterial, viral, or clinically diagnosed infection have been compared with those from patients without infection (*n* = 36)

Infection group:	Bacterial (*n* = 149)		Viral (*n* = 14)		Clinically diagnosed (*n* = 65)		LR test
Predictors at admittance	OR	95% CI	OR	95% CI	OR	95% CI	*p* value
Age per 10 years	1.17	(0.96, 1.44)	1.03	(0.63, 1.66)	1.01	(0.82, 1.25)	0.190
Female	1.79	(0.70, 4.54)	0.49	(0.08, 3.12)	1.02	(0.38, 2.70)	0.223
Duration of fever							0.073
I (<7 days)	1.00	Reference	1.00	Reference	1.00	Reference	
II (7–21 days)	1.55	(0.19, 12.35)	26.23	(0.29, 2408.69)	0.55	(0.06, 4.96)	
III (>21 days)	0.15	(0.01, 2.29)	49.68	(0.06, 40983.32)	0.50	(0.01, 23.54)	
IV (i.c.)	4.37	(0.23, 81.98)	0.32	(0.00, 30.10)	8.58	(0.32, 231.25)	
Temperature (°C)	1.94	(1.23, 3.06)	4.61	(1.76, 12.04)	1.69	(1.05, 2.72)	0.002
WBC count (×10^9^/L)	1.08	(0.99, 1.17)	1.00	(0.83, 1.20)	0.97	(0.89, 1.07)	0.003
CRP group							0.001
>100 mg/L	4.35	(0.92, 20.54)	0.24	(0.02, 3.82)	1.99	(0.38, 10.52)	
11–100 mg/L	1.45	(0.37, 5.74)	0.30	(0.04, 2.28)	2.78	(0.67, 11.56)	
0–10 mg/L	1.00	Reference	1.00	Reference	1.00	Reference	
MLR × Duration of fever^b^							<0.001
I (<7 days)	0.41	(0.03, 6.14)	0.00	(0.00, 0.34)	2.52	(0.43, 14.82)	
II (7–21 days)	11.87	(0.07, 1946.75)	0.00	(0.00, 0.03)	2.61	(0.05, 138.81)	
III (>21 days)	0.64	(0.01, 27.65)	0.00	(0.00, 471.25)	0.03	(0.00, 6079.60)	
IV (i.c.)	3.00	(0.53, 17.13)	0.41	(0.01, 16.82)	0.03	(0.00, 2.97)	

*LR* likelihood ratio, *OR* odds ratio, *CI* confidence interval, *WBC* white blood cell, *CRP* C-reactive protein, *i.c.* immunocompromised patients

^a^Altogether 299 patients were available for analysis, but 35 had missing data on one or more variables, leaving 264 patients for the multiple regression analysis

^b^Test of interaction: *p* = 0.001

**Fig. 2:**
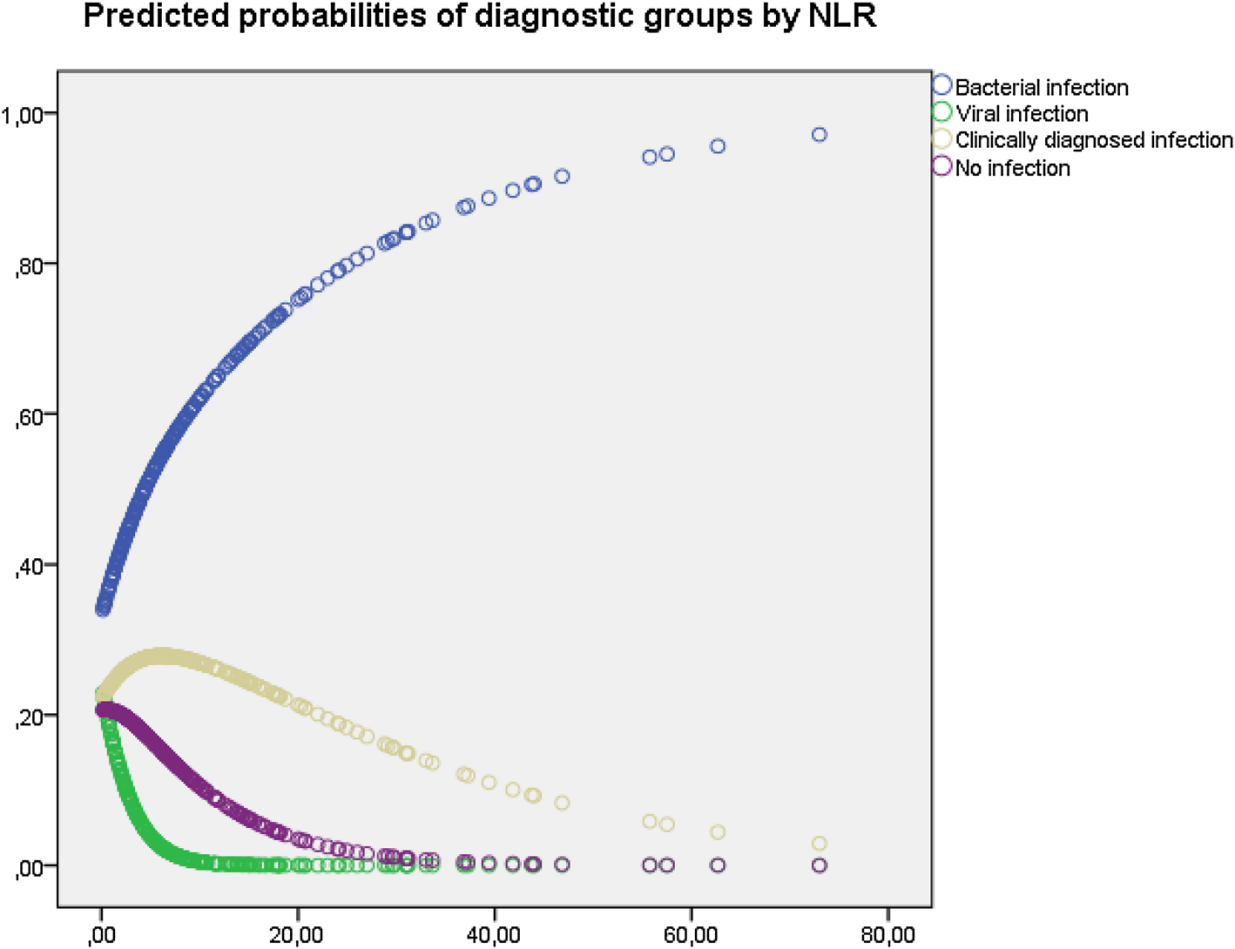
Predicted probabilities of diagnostic groups by neutrophil:lymphocyte ratio (NLR) based on an unadjusted multinomial regression model for 266 patients admitted for fever

**Fig. 3:**
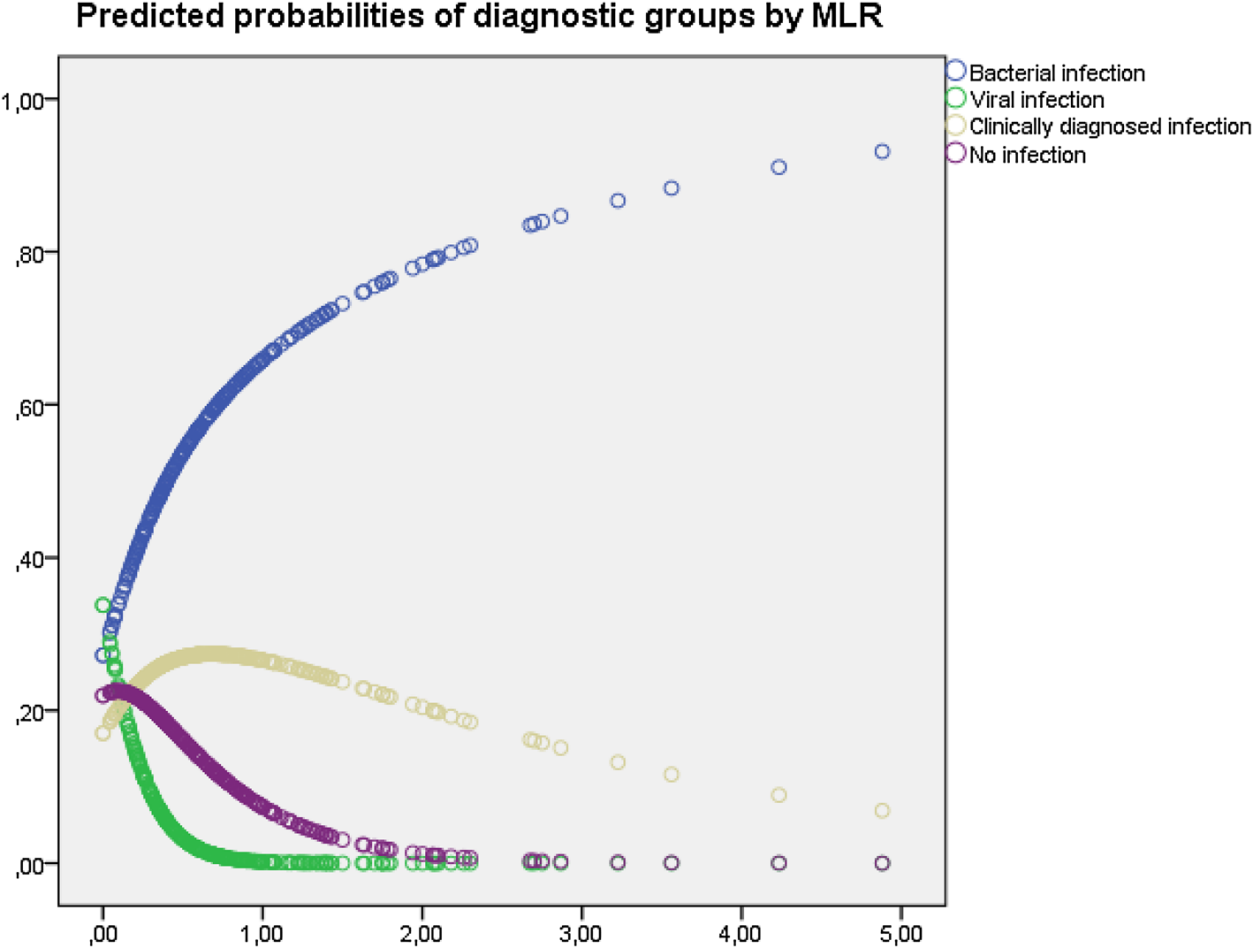
Predicted probabilities of diagnostic group by monocyte:lymphocyte ratio (MLR) based on an unadjusted multinomial regression model for 266 patients admitted for fever

Figures 2 and 3 show the unadjusted predicted probabilities from the multinomial logistic regression model of the four diagnostic groups according to NLR and MLR, respectively.

**Fig. 4:**
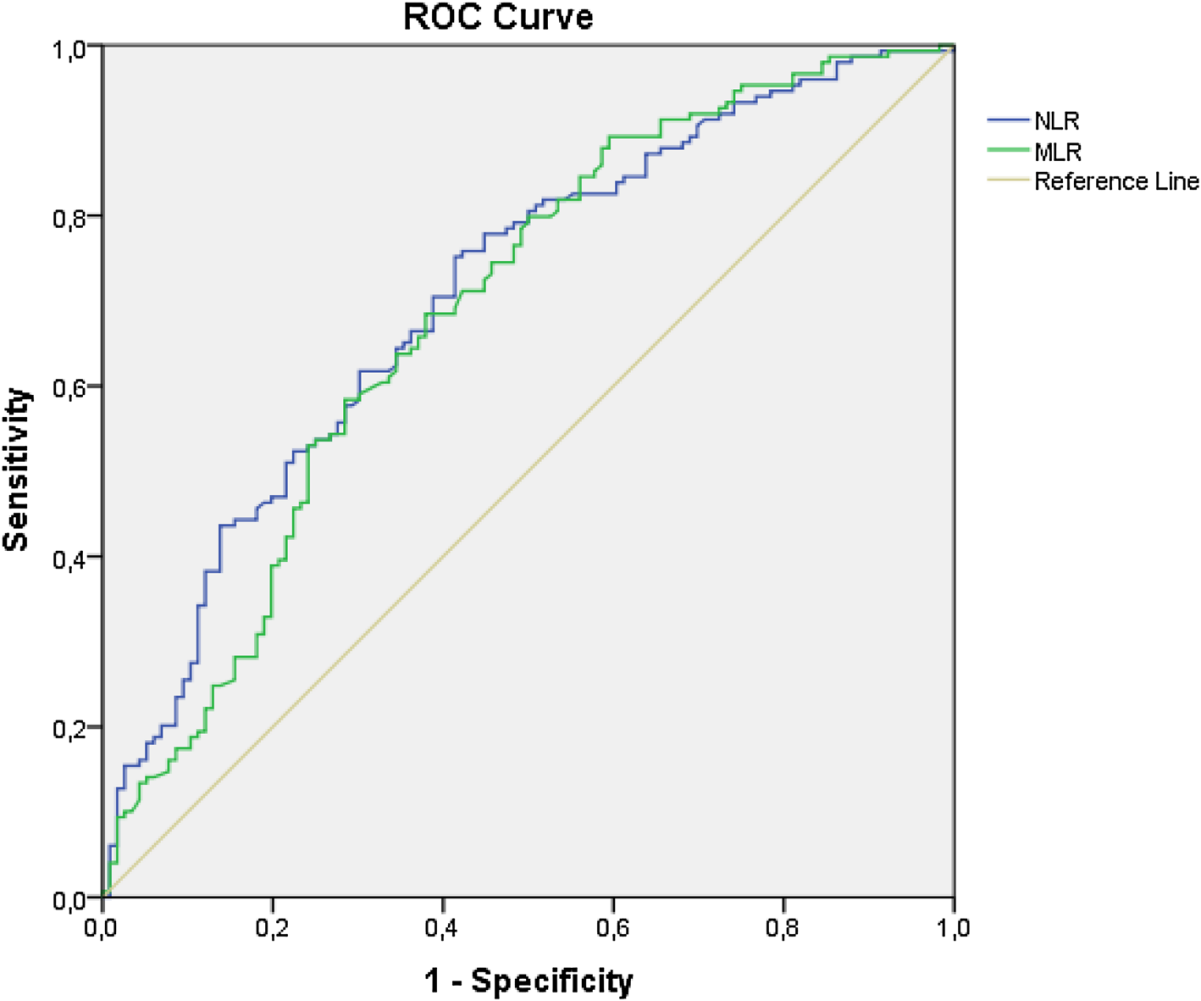
Receiver operating characteristic (ROC) curves for bacterial infection from 265 patients admitted for fever without diagnosis. Areas under the curves were 0.708 for the neutrophil:lymphocyte ratio (NLR) and 0.688 for the monocyte:lymphocyte ratio (MLR), respectively

Figure 4 shows the sensitivity and 1 − specificity of the NLR and MLR with respect to bacterial infection for both NLR and MLR.

## Discussion

Patients hospitalized for fever commonly represent diagnostic problems, and a correct diagnosis is, of course, required for adequate treatment. We have previously found the NLR to be higher in bacterial than in viral infection among patients hospitalized for fever. In that study, increased age gave significantly higher odds for bacterial infection, but gender was not a significant diagnostic factor [23]. In the present paper, we demonstrate that NLR and also MLR is higher in patients hospitalized for fever due to bacterial infection, and lower in those with viral infection, than in patients with non-infectious causes of fever (Table 1). This was more pronounced in patients with fever of less than one week’s duration (Table 2). Among patients with fever of less than one week’s duration, patients with septicemia had significantly higher NLR compared to patients with other bacterial infections (Table 3). The commonly used parameters to diagnose bacterial infection, such as WBC, neutrophils counts and CRP, did not differ significantly between septicemia and the other bacterial infection groups.

The NLR and MLR were highly correlated (Fig. 1), and the predicted probabilities of the different diagnostic groups by NLR (Fig. 2) and MLR (Fig. 3) showed great similarities. For example, a patient with NLR of nine has a predicted probability of having bacterial infection of 0.60 and viral of 0.01, but with a NLR of 33 these probabilities would be 0.85 and <0.01, and a patient with MLR of 1 has a predicted probability of having bacterial infection of 0.66 and viral of <0.01, but with MLR of two the predicted probability of having bacterial infection is 0.83 and viral <0.01, respectively.

Generally, higher values of NLR and MLR indicated larger probabilities for bacterial infection and low probabilities for viral infection (Fig. 4). This effect was especially pronounced in patients with fever less than 7 days at admission (Tables 3, 4, 5). For patients with low NLR and MLR, viral infection was more likely, except for immunosuppressed patients.

These observations indicate that the NLR and the MLR may be helpful in the differential diagnosis of patients with fever, and thus in deciding which patients should be considered for antibiotic therapy.

Several studies have shown increased NLR in infections [1–5, 25, 26], including meningitis [27]. The MLR has also been applied to this purpose [17–21]. However, none of these studies applied the ratios to discern between patients with fever due to infectious as opposed to non-infectious causes.

Not only infections, but a plenitude of other diseases has been associated with increased ratios, among these malignant and immunological diseases, conditions not uncommon among patients hospitalized for fever [11, 28–32]. Such patients were also present in our study, but they had ratios lower than patients with bacterial and higher than patients with viral infection.

In a study of 1468 patients with suspected bacteremia and septicemia, using procalcitonin (PCT) as a reference, Gürol et al. [33] found NLR to have higher sensitivity than CRP and WBC. They suggested the following intervals for local infection [5–10), systemic infection [10–13), septicemia [13–15), and for septic shock at least 15, respectively.

These authors thus found NLR as a more convenient marker for infection than CRP, with a high specificity (83.9%) but a moderate sensitivity for diagnosing septicemia in critically ill patients.

Although the patient groups are very dissimilar, the suggested cutoff values of Gürol et al. [33] correspond reasonably well to the results of the present study.

However, although Lowsby et al. [25] found NLR to outperform conventional markers of infection, including WBC count, PMN count, and CRP, it was insufficient in itself to guide clinical management of patients with suspected blood stream infection. In addition, the ratios may vary according to the course of the disease, as Riché et al. [7] found the NLR to be reversed in early versus late death from septic shock, and Tannverdi et al. [26] found PCT better for predicting bacterial infection than the CRP level or the NLR.

For such reasons, some authors, in particular Nuutila et al. [34] have applied a variety of indices to diagnose bacterial infections.

However, as opposed to NLR and MLR, these indices employ tests not commonly performed in routine laboratories, and may thus be unavailable to many clinicians.

Blot et al. [35], using a leukocyte score with points for neutropenia, lymphopenia and monocytopenia, found a high score to be significantly associated with mortality in bacteremic pneumococcal pneumonia, but this score has to our knowledge not been applied to other groups of patients.

Our study is small and retrospective. However, all patients admitted for fever were followed prospectively until the final diagnosis. The results indicate that NLR and MLR may be useful in the differential diagnosis of patients hospitalized for fever, and may be helpful in deciding which patients hospitalized for fever have a greater likelihood for bacterial infection and should be considered for antibiotic treatment. Patients with septicemia had significantly higher NLR compared to patients with other bacterial infections with fever for less than one week. The commonly used parameters to diagnose bacterial infection, such as WBC, neutrophils counts and CRP, did not differ significantly between septicemia and the other bacterial infection groups. We conclude that NLR is a more useful diagnostic tool to identify patients with septicemia, the most serious bacterial infection, than other more commonly used diagnostic blood tests.
